# High-efficiency lysis of cervical cancer by allogeneic NK cells derived from umbilical cord progenitors is independent of HLA status

**DOI:** 10.1007/s00262-016-1919-1

**Published:** 2016-10-25

**Authors:** John P. Veluchamy, A. Marijne Heeren, Jan Spanholtz, Jaap D. H. van Eendenburg, Daniëlle A. M. Heideman, Gemma G. Kenter, Henk M. Verheul, Hans J. van der Vliet, Ekaterina S. Jordanova, Tanja D. de Gruijl

**Affiliations:** 1 Department of Medical Oncology, VU (Vrije Universiteit) University Medical Center-Cancer Center Amsterdam, De Boelelaan 1117, 1081 HV Amsterdam, The Netherlands; 2 Glycostem Therapeutics, Oss, The Netherlands; 3 Department of Obstetrics and Gynecology, Center Gynecological Oncology Amsterdam (CGOA), VU University Medical Center, De Boelelaan 1117, 1081 HV Amsterdam, The Netherlands; 4 Department of Pathology, Leiden University Medical Center, Albinusdreef 2, 2333 ZA Leiden, The Netherlands; 5 Department of Pathology, VU University Medical Center, De Boelelaan 1117, 1081 HV Amsterdam, The Netherlands

**Keywords:** Cervical cancer, Peripheral blood NK cells, Umbilical cord blood stem cell-derived NK cells, CET, NK ligands and receptors, Adoptive NK immunotherapy

## Abstract

Down-regulation of HLA in tumor cells, low numbers and dysfunctionality of NK cells are commonly observed in patients with end-stage cervical cancer. Adoptive transfer of high numbers of cytotoxic NK cells might be a promising treatment approach in this setting. Here, we explored the cytotoxic efficacy on ten cervical cancer cell lines of activated allogeneic NK cells from two sources, i.e., peripheral blood (PBNK) with and without cetuximab (CET), a tumor-specific monoclonal antibody directed against EGFR, or derived from umbilical cord blood (UCB-NK). Whereas CET monotherapy was ineffective against the panel of cervical cancer cell lines, irrespective of their EGFR expression levels and despite their *RAS* ^wt^ status, it significantly enhanced the in vitro cytotoxic efficacy of activated PBNK (*P* = 0.002). Equally superior cytotoxicity over activated PBNK alone was achieved by UCB-NK (*P* < 0.001). Both PBNK- and UCB-NK-mediated cytotoxic activity was dependent on the NK-activating receptors natural killer group 2, member D receptor (NKG2D) and DNAX accessory molecule-1 (DNAM-1) (*P* < 0.05) and unrelated to expression levels of the inhibitory receptors HLA-E and/or HLA-G. Most strikingly, whereas the PBNK’s cytotoxic activity was inversely correlated with HLA-ABC levels (*P* = 0.036), PBNK + CET and UCB-NK cytotoxicity were entirely independent of HLA-ABC expression. In conclusion, this study provides a rationale to initiate a clinical trial for cervical cancer with adoptively transferred allogeneic NK cells, employing either UCB-NK or PBNK + CET for EGFR-expressing tumors. Adoptive transfer of UCB-NK might serve as a generally applicable treatment for cervical cancer, enabled by HLA-, histology- and HPV-independent killing mechanisms.

**Electronic supplementary material:**

The online version of this article (doi:10.1007/s00262-016-1919-1) contains supplementary material, which is available to authorized users.

## Introduction

Persistent infection of the cervical epithelium by high-risk HPV can lead to cervical intraepithelial neoplasia which may progress to invasive cervical cancer, such as squamous cell carcinoma, adenosquamous cell carcinoma or adenocarcinoma [1–3].

Treatment for cervical cancer includes conventional surgery, chemotherapy and/or radiation. In addition, in advanced (metastatic) disease, targeted therapies are widely explored. Unfortunately, targeted intervention strategies using small molecules, angiogenesis inhibitors and monoclonal antibodies directed against specific tumor antigens and proliferation pathways have had limited success in restricting cervical tumor growth so far [4, 5]. In cervical cancer, EGFR is variably expressed in 80 % of the tumor tissues [6]. Overexpression of EGFR has been associated with poor prognosis in cervical cancer, making EGFR an obvious candidate for therapeutic targeting [7, 8]. However, treatment with cetuximab (CET) (chimeric IgG_1_, anti-EGFR mAb) as monotherapy or CET in combination with chemotherapy was ineffective in patients with cervical cancer, in spite of the apparent absence of activating mutations in the EGFR pathway [9, 10].

Immunotherapy of cervical cancer has been clinically explored with limited success. Efforts so far have mostly focused on vaccination approaches against HPV-derived oncogenes (E6 and E7) to trigger an efficacious antitumor T cell response [11]. Failure to improve clinical outcome may at least in part be due to extensive HLA down-regulation commonly observed in cervical cancer [12, 13] (Heeren et al. 2015, submitted). In these cases, NK cell-based therapies may prove more effective than T cell-based approaches. Indeed, the role of the innate immune response in host defense and viral clearance during (early) infection is well recognized [14]. NK cells are potent in exerting rapid cytotoxicity by releasing cytotoxic granzyme B and perforin in order to lyse virus-infected cells and tumor cell targets. Functional activity of NK cells is regulated by an equilibrium between inhibitory (e.g., CD94/NKG2A) and activating (e.g., CD16, DNAM-1, CD94/NKG2C, CD94/NKG2D) receptors [15, 16].

Infiltrating NK cells are observed in low-grade and high-grade cervical intraepithelial neoplasia lesions and to a lesser extent in cervical carcinoma [13, 17–20]. In vitro studies have shown that peripheral blood NK cells (PBNK) are able to kill HPV-infected cell lines [18, 20, 21]. However, NK cells are often dysfunctional and low in number in cervical cancer patients and thereby unable to mount efficient cytotoxicity against tumors [22, 23]. NK cytotoxic function is also counteracted by several cervical tumor escape mechanisms, including low expression of activating NK cell receptor ligands (e.g., MICA/B, ULBPs, Nectin, PVR) and aberrant expression of suppressive non-classical HLA molecules (e.g., HLA-E and -G) by tumor cells [18, 24–26] (Heeren et al. 2016, submitted). Ex vivo expanded autologous NK cells, adoptively transferred for the treatment of solid tumors, in most studies have yielded disappointing results, underscoring the dire need for the development of more powerful therapeutic approaches to overcome tumor-associated NK cell dysfunctionality and the inherent resistance to cytolysis of cancer cells. Clinical studies exploring the use of ex vivo expanded allogeneic PBNK from healthy donors also yielded low antitumor efficacy [27, 28], which may have been due to their limitations in terms of cell yield, purity, ability to expand in vivo and cytotoxic capacity [29].

An attractive alternative approach would be the use of umbilical cord blood CD34^+^ stem cell-derived NK cells (UCB-NK), which are feeder cell-free cultures that can be differentiated and efficiently expanded up to 10,000-fold, resulting in a highly pure product with a high cytolytic capacity [30]. Yet another alternative might be to enhance PBNK cell-mediated cytolysis of cervical tumor cells by the tumor-targeted IgG1 monoclonal antibody CET, to invoke antibody-dependent cell-mediated cytotoxicity (ADCC) [31].

In this comparative study, we explored the antitumor efficacy of two clinically applicable therapeutic strategies, i.e., UCB-NK versus allogeneic PBNK + CET, for cervical cancer. Of note, the combination with CET is not a viable option for UCB-NK as in vitro they do not express sufficient levels of the required Fc receptor CD16 to obtain functional benefit [32] (Veluchamy et al., manuscript in preparation). A series of in vitro NK cytotoxicity assays was conducted to compare antitumor potency of PBNK from healthy volunteers, with or without co-incubation with CET with that of umbilical cord blood-derived NK cell (UCB-NK) monotherapy against various cervical cancer cell lines. These cell lines (*n* = 10) were stratified based on infection with different HPV types, histological origins and differential expression levels of NK-activating and inhibitory ligands. The findings from this preclinical study strongly support the use of allogeneic UCB-NK derived from umbilical cord precursor cells and outline the advantages of their use as monotherapy in the treatment of cervical cancer.

## Materials and methods

### Cell lines

Cervical cancer cell lines CSCC7, CC8, CC10A, CC10B, CC11A and CC11B were generated in the Department of Pathology of Leiden University Medical Center (The Netherlands) from primary tumors as described previously [33]. These patient-derived cell lines as well as commercially obtained cervical cancer-derived cell lines, HeLa, SiHa, CaSki and C33A (American Type Culture Collection) were maintained in DMEM (Lonza) medium containing 4.5 g/L glucose, 10 % FCS (Hyclone), 10 µg/mL gentamicin and 0.25 µg/mL amphotericin B (Gibco), 100 units penicillin/100 units streptomycin/0.3 mg/mL glutamine (Thermo Fisher Scientific). Cell cultures were maintained at 37 °C in a humidified atmosphere containing 5 % CO_2_. See Table 1 for cell line characteristics.

**Table 1:** Cell line characteristics

Cell line	Histology^a^	HPV type^a^	*RAS* status^b^
HeLa	AC	18	Wild type
SiHa	SCC	16	Wild type
CaSki	Epidermoid	16	Wild type
C33A	SCC	Negative	Wild type
CSCC7	SCC	16	Wild type
CC8	ASC	45	Wild type
CC10A	AC	45	Wild type
CC10B	AC	45	Wild type
CC11A	AC	67	Wild type
CC11B	SCC	67	Wild type

*AC* adenocarcinoma, *SCC* squamous cell carcinoma, *ASC* adenosquamous carcinoma

^a^Characteristics adapted from www.lgcstandards-atcc.org and [33]

^b^ *RAS* status obtained from www.lgcstandards-atcc.org and *RAS* typing performed by the RAIDs FP7 Consortium and in own institute

### Phenotyping of cervical cancer cell lines

To phenotype cervical cancer cell lines, cell suspensions in PBS supplemented with 0.1 % BSA and 0.02 % NaN_3_ (FACS buffer) were stained for 30 min at 4 °C using antibodies to HLA-ABC (clone w6/32, Immunotools) (labeled with FITC), HLA-E (clone 3D12HLA-E, eBioscience), HLA-G (clone 87G, Biolegend), EGFR (clone EGFR.1, BD Biosciences), PVR (clone SK11.4, Biolegend), MICA/B (clone 6D4, Biolegend), ULBP2/5/6 (clone #165903, R&D systems), ULBP1 (clone #170818, R&D systems) and ULBP3 (clone #166510, R&D systems) (all labeled with PE). IgG_1_, IgG_2a_ and IgG_2b_ isotype antibodies were used as negative controls. After incubation, the cells were washed with FACS buffer and analyzed using a flow cytometer LSR Fortessa (BD Biosciences). Phenotypic analyses were obtained from at least two independent experiments performed on each cell line. Data were analyzed using Kaluza software (Beckman coulter) and calculated as specific (geometric) mean fluorescence intensity (MFI) (MFI; geometric mean fluorescence of marker − geometric mean fluorescence of isotype).

### *RAS* typing

*RAS* status was obtained from rational molecular assessments and innovative drugs selection (RAIDs) project data (http://www.raids-fp7.eu/project-overview.html) and www.lgcstandards-atcc.org for cell lines HeLa, SiHa, CaSki, C33A, CSCC7, CC10A and CC10B. In addition, full *RAS* typing (i.e., *BRAF* exon 15, *KRAS* exon 2–4 and *NRAS* exon 2–4) was performed for cell lines CC8, CC11A and CC11B at the molecular pathology lab of the Department of Pathology of the VU University Medical Center (Amsterdam, The Netherlands) using high-resolution melting assay followed by Sanger sequencing of using high-resolution melting PCR products with an aberrant melt curve, essentially as described previously [34, 35].

### PBMC isolation and NK cell isolation

Whole blood samples from four healthy volunteers were collected. PBMC were isolated using Lymphoprep™ (STEMCELL Technologies, The Netherlands) density gradient centrifugation. CD56^+^ NK cells were isolated from PBMC using a MACS^®^ Human NK cell isolation kit (Miltenyi Biotech, Bergisch Gladbach, Germany) according to the manufacturer’s instructions. The cell number and purity of the isolated PBNK was analyzed by flow cytometry. Isolated NK cells were activated overnight with 1000 U/mL IL-2 (Proleukin^®^; Chiron, München, Germany) and 10 ng/mL IL-15 (CellGenix) before use in cytotoxicity assays. NK cell purity and viability were checked by flow cytometry using the following antibodies: 7-aminoactinomycin D (7AAD; Sigma-Aldrich), CD3 (labeled with VioBlue), CD56 (labeled with APC-Vio770) and CD16 (labeled with APC) (all from Miltenyi Biotech). Purity of NK cells obtained from NK donors was 87 ± 6 %. For cytotoxicity assays, only PBNK with CD16 expression rates exceeding 80 % were used.

### UCB-NK isolation and cultures

Allogeneic NK cells were generated from cryopreserved umbilical cord blood hematopoietic stem cells as previously described [36]. CD34^+^ UCB cells (3 × 10^5^ mL) were plated into 12-well tissue culture plates (Corning Incorporated, Corning, NY) in Glycostem Basal Growth Medium (GBGM^®^) (Clear Cell Technologies, Beernem, Belgium) supplemented with 10 % human serum (Sanquin Bloodbank, The Netherlands), 25 ng/mL of SCF, Flt-3L, TPO and IL-7 (CellGenix, Germany). In the expansion phase II, from day 9 to 14, TPO was replaced with 20 ng/mL IL-15 (CellGenix). During the first 14 days of culture, low molecular weight heparin (LMWH) (Clivarin^®^; Abbott, Wiesbaden, Germany) in a final concentration of 20 µg/mL and a low-dose cytokine cocktail consisting of 10 pg/mL GM-CSF (Neupogen), 250 pg/mL G-CSF and 50 pg/mL IL-6 (CellGenix) were added to the expansion cultures. Cells were refreshed with new medium twice a week and maintained at 37 °C, 5 % CO_2_. On day 14, the NK cell differentiation process was initiated by addition of NK cell differentiation medium consisting of the same basal medium with 2 % human serum but with high-dose cytokine cocktail consisting of 20 ng/mL of IL-7, SCF, IL-15 (CellGenix) and 1000 U/mL IL-2 (Proleukin^®^; Chiron, München, Germany). Cultures were refreshed every 2–3 days and maintained till day 35. For cytotoxicity assays, UCB-NK was used with CD56^+^ cells >85 % purity.

### In vitro NK cytotoxicity assays

Cervical cancer cell lines (target cells) were labeled with 5 µM pacific blue succinimidyl ester (PBSE; Molecular Probes Europe, Leiden, The Netherlands) in a concentration of 1 × 10^7^ cells/mL for 15 min at 37 °C. After incubation, cells were washed and resuspended in DMEM culture medium to a final concentration of 1 × 10^6^ mL. PBNK and UCB-NK were washed with PBS and also resuspended in GBGM medium to a final concentration of 1 × 10^6^ mL. Target cells were co-cultured in triplicate with effector cells (PBNK or UCB-NK), with or without 5 µg/mL CET at an *E*:*T* ratio of 1:1 in a total volume of 100 µL in FACs tubes (5 × 10^4^ targets in 50 µL of DMEM culture medium incubated with 5 × 10^4^ effectors in 50 µL of GBGM medium). PBNK, UCB-NK and target cells alone were cultured in triplicate as controls. To measure degranulation by PBNK and UCB-NK, anti-CD107a PE (Miltenyi Biotech, Germany) was added at the beginning of the assay. After incubation for 4 h at 37 °C, cells were harvested and stained with 7AAD, CD56 (labeled with APC-Vio770) and CD16 (labeled with APC) (all from Miltenyi Biotech, Germany). For NK flow cytometry and blocking experiments, NKG2D PE (clone ON72, Beckman Coulter) and DNAM-1 FITC (clone DX11, BD Pharmingen™) were used at 10 µg/mL. Further, killer-cell immunoglobulin-like receptor 2D (PanKIR2D), FITC (clone NKVFS1) and CD94/NKG2A PE-Vio770 (clone REA110) (both from Miltenyi Biotech) were used to screen inhibitory receptor expression on PBNK and UCB-NK. BD LSR Fortessa™ was used for readout of the cytotoxicity assays. Data were analyzed using Kaluza software (Beckman coulter). Percentages of specific NK degranulation were calculated as ∆CD107a^+^ NK cells [i.e., (target cells + NK cells) – (NK cells only)] and percentages of cytotoxicity as ∆7AAD^+^ target cells [i.e., (target cells + NK cells) – (target cells only)]. See Supplementary Figure 1 for a representative gating example.

### Statistical analysis

Statistical analysis was performed using GraphPad Prism software. Statistical significance of differences between conditions were determined using a parametric paired *t* test, unpaired *t* test or a one-way ANOVA with Bonferroni’s multiple comparison test and a two-way ANOVA with multiple comparisons between column means. Correlation analyses between percentages NK degranulation, cytotoxicity and MFI were performed using Pearson’s analysis. A *P* value of <0.05 was considered statistically significant.

## Results

### Comparative analysis of NK cell cytotoxic activity against cervical cancer cell lines

**Fig. 1:**
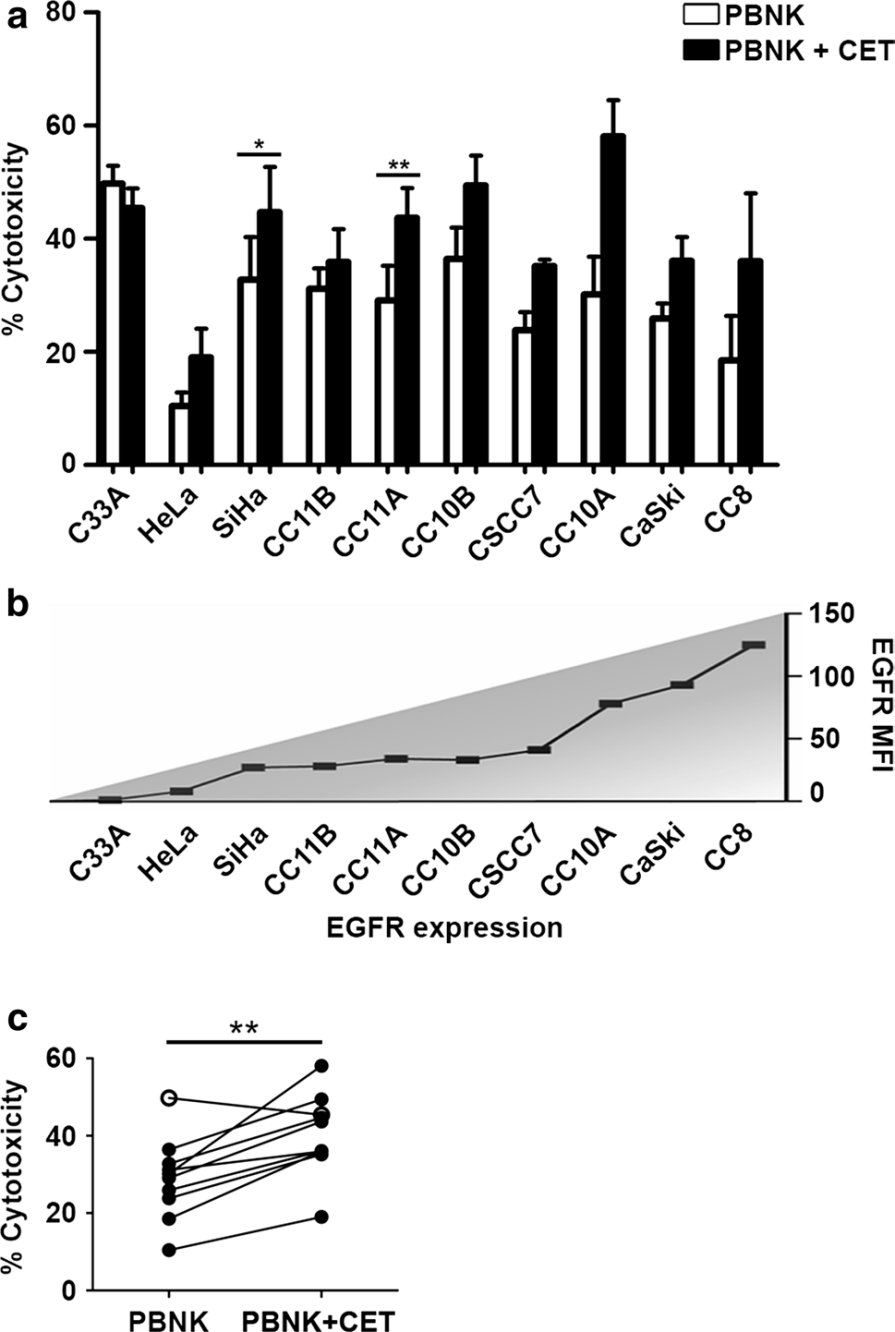
PBNK cytotoxicity against cervical cancer cells alone and in combination with CET. **a** Cytotoxicity levels (Δ7AAD) of activated PBNK (*open bars*) and PBNK + cetuximab (CET) (*closed bars*) against ten cervical cancer cell lines, **b** arranged in order of EGFR expression level. *Bars* are means of triplicate values from four experiments with four different PBNK donors for C33A, HeLa, SiHa, CC11B, CC11A, CC10B, CC10A, CaSki and two experiments with two different PBNK donors for CSCC7 and CC8. *Bars* represent mean ± SEM. **c** Significantly higher cytotoxicity levels (Δ7AAD) were observed in all cell lines after co-culture with PBNK + CET compared to PBNK, except for C33A (*open circle*). *P < 0.05 and **P < 0.01 calculated with paired *t* test

Initially, we compared the antitumor potency of healthy activated PBNK in the presence or absence of CET. Ten cervical cancer cell lines (EGFR-expressing except for C33A, and all *RAS* ^wt^; Table 1) were subjected to PBNK only, CET only, or to a combination of PBNK with CET in order to examine ADCC effects. In line with previous studies, CET as monotherapy did not induce cell death in any of the cell lines tested (data not shown). However, cervical cancer cell lines were sensitive in varying degrees to PBNK-induced cell lysis (Fig. 1a), independent of their EGFR expression levels (Fig. 1b), with consistently and significantly higher cytotoxicity rates when coated with CET (*P* = 0.002) (Fig. 1c). C33A (EGFR-negative cell line) was the only cell line that did not display a higher cytotoxicity rate when PBNK were combined with CET (Fig. 1a–c).

**Fig. 2:**
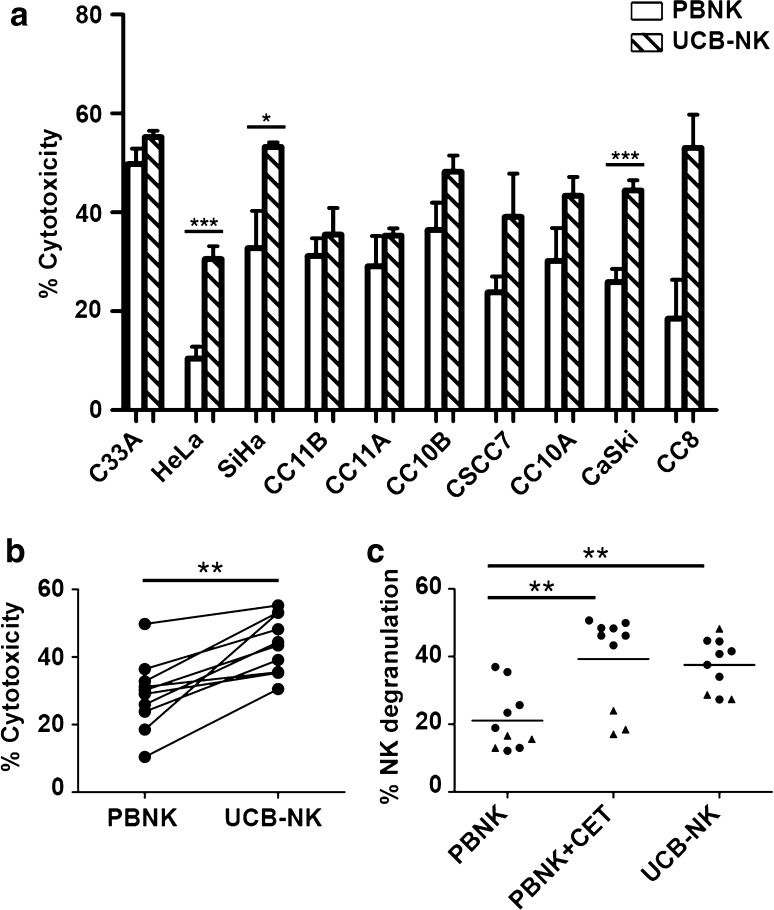
PBNK and UCB-NK cytotoxicity against cervical cancer cells. **a** Cytotoxicity levels (Δ7AAD) of PBNK (*open bars*) and UCB-NK (*hatched bars*) against ten cervical cancer cell lines. *Bars* are means of triplicate values from four experiments with four different PBNK donors for C33A, HeLa, SiHa, CC11B, CC11A, CC10B, CC10A, CaSki and two experiments with two different PBNK donors for CSCC7 and CC8 and five experiments for UCB-NK using five different UCB-NK donors for all cell lines; *Bars* represent mean ± SEM. PBNK data used to compare with UCB-NK in **a** are the same dataset as Fig. 1a. **b** Significantly higher cytotoxicity levels (Δ7AAD) were observed in all cell lines after co-culture with UCB-NK compared to PBNK. **c** Significantly higher levels of NK degranulation (ΔCD107a) in PBNK + cetuximab (CET) and UCB-NK conditions compared to PBNK only condition. *Triangles* denote cell lines with low EGFR levels, i.e., C33A, HeLa and SiHa. *P < 0.05, **P < 0.01 and ***P < 0.001 calculated in A and B with unpaired *t* test, in C with one-way ANOVA, Bonferroni’s multiple comparison test

Next, activated PBNK were compared with UCB-NK for their ability to induce target cell death. UCB-NK was significantly more cytotoxic than PBNK, consistently inducing higher rates of tumor cell death in all tested cell lines (*P* < 0.001) (Fig. 2a, b). Note that the PBNK cytotoxicity data presented in Fig. 2a are the same as those in Fig. 1a. The cytotoxicity levels were similar for UCB-NK and PBNK + CET (Figs. 1a, 2a). This was further borne out by observed degranulation levels of NK cells in response to exposure to the cervical cancer cell lines, as measured by CD107a surface expression. These were comparably and significantly elevated in the PBNK + CET and UCB-NK conditions over PBNK alone (Fig. 2c, Supplementary Figure 2). UCB-NK were not tested in combination with CET due to their low surface expression of CD16a, which is essential for ADCC in combination with therapeutic mAbs (data not shown). Interestingly, PBNK degranulation levels were low in combination with CET upon exposure to cervical cancer cell lines expressing low levels of EGFR (C33a, HeLa and SiHa: denoted in Fig. 2c by triangles). In contrast, degranulation levels in UCB-NK were generally high. PBNK, PBNK + CET and UCB-NK cytotoxicity levels per histological subtype and HPV type of cervical cancer cell lines are shown in Supplementary Figure 3. It shows that irrespective of HPV or histological tumor type, highest cytotoxicity was consistently achieved by UCB-NK.

### Expression of NK-activating receptors and their ligands and their contribution to mediating cytotoxicity

**Fig. 3:**
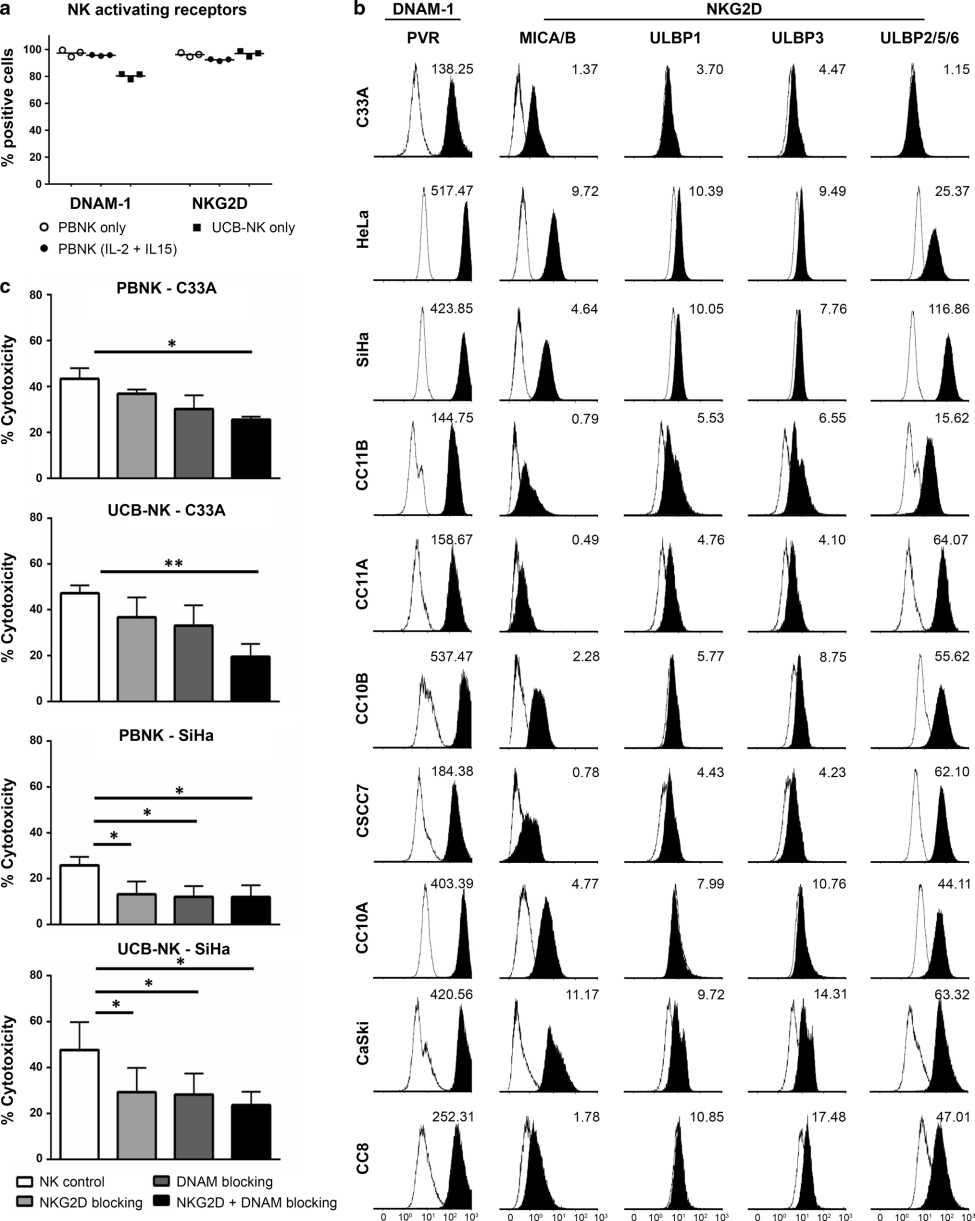
NK-activating receptors in PBNK and UCB-NK and their ligand expression in cervical cancer cell lines and their influence on NK cytotoxicity. **a** Percentage of positive cells within the NK cell population for NK-activating receptors DNAM-1 and NKG2D for PBNK only, PBNK stimulated with cytokines (IL-2 + IL-15) and UCB-NK only were determined by flow cytometry. The data presented is from three representative donors for PBNK and UCB-NK. PBNK only are denoted by *open circles*, PBNK (IL-2 + IL-15) are denoted by *closed circles* and UCB-NK only by *closed squares*. **b** Representative example of histograms showing geometric mean fluorescence intensity (MFI) for NK-activating ligands PVR (ligand of DNAM-1 receptor), MICA/B and ULBP1, −3 and −2/5/6 (ligands of NKG2D receptor). **c** PBNK and UCB-NK were coated with NKG2D and/or DNAM-1 blocking antibodies and incubated with C33A and SiHa cells. Cytotoxicity levels (Δ7AAD) were measured from 7AAD + C33A and SiHa cells at the end of a 4 h assay. Data presented are means of triplicate values from three different PBNK and three different UCB-NK donors; *Bars* represent mean ± SEM. *P < 0.05 and **P < 0.01 calculated with paired, two-way ANOVA multiple comparisons of column means

To investigate the involvement of activating receptors in mediating the cytotoxic activity of PBNK and UCB-NK, the expression of the two major NK-activating receptors DNAM-1 and NKG2D on the NK cells described to be involved in the recognition of cervical cancer cells, and their respective ligands, i.e., PVR and MICA/B, ULBPs, on the tested cervical cancer cell lines, were assessed. Similarly, high levels of DNAM-1 and NKG2D were observed on both PBNK and UCB-NK (Fig. 3a). The cell lines showed differential expression of the NK-activating ligands, but all were positive for PVR, the DNAM-1 ligand, and at least one of the NKG2D ligands (Fig. 3b). From the panel of cell lines, SiHa (with highest expression levels of PVR and ULBP-2/5/6) and C33A (with lowest expression levels of PVR and ULBP-2/5/6) were selected as target cells in functional blocking studies. The relatively low ligand expression levels on C33A required combined blocking of DNAM-1 and NKG2D to achieve a significant reduction in either PBNK- or UCB-NK-mediated cytotoxicity (Fig. 3b). In contrast, blocking either DNAM-1 or NKG2D already led to significant reductions of cytotoxicity in SiHa cells (Fig. 3c). These data show dependence (at least in part) of both PBNK and UCB-NK on DNAM-1 and NKG2D for their cytotoxic potency.

### Differential expression of NK inhibitory receptors and their ligands in relation to level of cytolysis

**Fig. 4:**
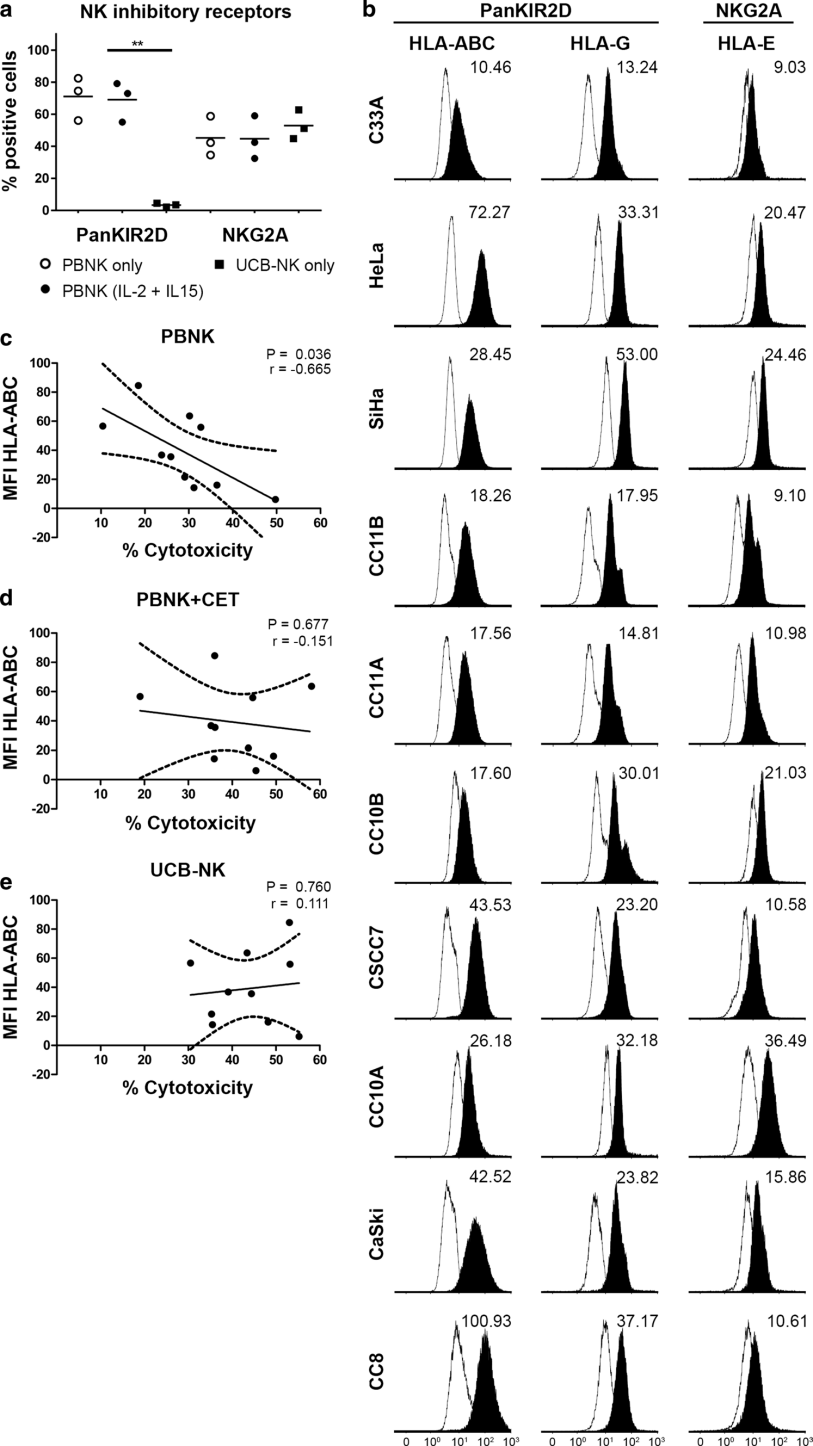
Effects of NK inhibitory ligands on NK cytotoxicity against cervical cancer cells. **a** Percentage of positive cells within the NK cell population for NK inhibitory receptors KIR2D and CD94/NKG2A for PBNK only, PBNK stimulated with cytokines (IL-2 + IL-15) and UCB-NK only were determined by flow cytometry. The data presented is from three representative donors for PBNK and UCB-NK. PBNK only are denoted by *open circles*, PBNK (IL-2 + IL-15) are denoted by *closed circles* and UCB-NK by *closed squares*. **b** Representative *histogram plots* showing geometric mean fluorescence intensity (MFI) of NK inhibitory ligands HLA-ABC, HLA-E and HLA-G on cervical cancer cells; representative *plots* of 2–3 separate analyses are shown. Correlation analysis of MFI of HLA-ABC with % cytotoxicity (Δ7AAD) by **c** PBNK, **d** PBNK + cetuximab(CET) and **e** UCB-NK. *Dotted lines* represent 95 % confidence interval of the regression line. Four experiments with four different PBNK donors for C33A, HeLa, SiHa, CC11B, CC11A, CC10B, CC10A, CaSki, two experiments with two different PBNK donors for CSCC7 and CC8 and five experiments with five different UCB-NK donors were used for this experiment. *P* value was calculated with Pearson’s analysis

To investigate the effect of NK inhibitory receptors on the observed cytotoxic efficacy, the expression levels of KIR2D and NKG2A on the NK cells, and of their respective ligands, i.e., HLA-ABC/-G and HLA-E [37], on the cervical cancer cell lines, were assessed (Fig. 4a, b). Irrespective of overnight activation with IL-2/IL-15, PBNK expressed high levels of both KIR2D and NKG2A, whereas UCB-NK only expressed equivalent levels of NKG2A, but no KIR2D. All classical and non-classical HLA molecules were expressed on all ten cervical cancer cell lines, but in widely varying degrees (Fig. 4b). Correlation analyses showed a relationship only between HLA-ABC expression levels and levels of cytotoxicity achieved by PBNK, with lower HLA-ABC levels allowing for higher levels of cytotoxicity (*P* = 0.036, Fig. 4c). In contrast, PBNK + CET (Fig. 4d) and UCB-NK cytotoxicity were totally independent of HLA-ABC expression levels (Fig. 4e). No other correlations were found between cytotoxicity levels and HLA-E or HLA-G expression levels on cervical cancer cell lines (data not shown).

## Discussion

Cervical cancer is the fourth most common malignancy in women worldwide. Survival is severely reduced in case of lymph node metastasis, with no curative treatment options available. In cervical cancer, ACT involving T cell or NK cell-based therapies has not yet been widely explored, but they have been successfully applied in the treatment of various other cancer types [38–40]. In one clinical trial, adoptive transfer of tumor-infiltrating T cells in metastatic cervical cancer resulted in tumor responses in 3/9 patients with complete remission in 2/9 patients [41]. These findings suggest that ACT could be a viable treatment option for some patients with cervical cancer. However, most cervical tumors have HLA class I alterations and will therefore not respond completely to T cell-based therapies [13, 42] (Heeren et al., submitted). NK cell-based therapies present a viable alternative in that case, but in advanced cervical cancer, these effector cells are often impaired in their functionality [23, 24]. In this study, we therefore explored the possible therapeutic efficacy of allogeneic NK cells. Clinically applicable NK cells may be derived from two sources, i.e., NK cells derived from peripheral blood and NK cells cultured and expanded from umbilical cord blood stem cells. We tested their cytotoxic efficacy (with and without CET for PBNK) on ten cervical cancer cell lines representing different histological subtypes, HPV types and expressing differential levels of NK-activating and inhibitory ligands.

Initially, we investigated the effect of PBNK alone and a combination of PBNK with CET on the cervical cancer cell lines. From the literature, it is known that cervical tumors often present with variable levels of EGFR [6, 8]. In colorectal cancers, mutant KRAS is associated with resistance to CET [43]. Although most of the cervical cancer cell lines were EGFR positive and all were *RAS* ^wt^, their EGFR expression levels were relatively low, and, in keeping with clinical observations for cervical cancer, they did not respond to CET as a single agent [9, 10, 44]. Our observation of increased PBNK cytotoxicity upon combination with CET is in line with a report by Meira et al. [45] who showed that one of the antitumor effector mechanisms upon combined CET and chemoradiation actually was ADCC.

Next, we compared the functionality of PBNK with that of ex vivo generated UCB-NK derived from cord blood stem cells and showed that UCB-NK were significantly more cytotoxic than PBNK (Fig. 2). NK cytotoxicity and NK degranulation levels were equivalent for UCB-NK and PBNK + CET. Further study of the NK killing mechanism revealed that the cytotoxic activity of both PBNK and UCB-NK was at least in part dependent on DNAM-1 and NKG2D receptors, as also previously reported for an NK cell line (NKL) and cytotoxicity it induced in the CaSki and SiHa cell lines [18]. This was in keeping with high expression levels of both NKG2D and DNAM-1 observed on both PBNK and UCB-NK. As complete abrogation of tumor cell killing was not achieved by combined blocking of DNAM-1 and NKG2D on activated PBNK and UCB-NK, other NK killing mechanisms such as NKp44/NKp44L, TRAIL (tumor necrosis factor-related apoptosis-inducing ligand) and FAS (Fas ligand interactions) also might contribute to the observed target cell lysis [46, 47]. Indeed, NKp44 has been previously reported as highly expressed on expanded UCB-NK, in sharp contrast to PB-NK cells, which in the steady state do not express NKp44 [32]. The known ligands for NKp44 have mostly been associated with microbial responses, whereas the identity of cancer-associated ligands until recently has remained mostly obscure. A ligand for NKp44 has now been identified on tumor cells, designated NKp44L, which opens the way for further exploration of the relative importance of this activating receptor axis in NK-mediated tumor cytolysis [48].

Interestingly, in the present study, we have shown the predominant effect of HLA class I expression on the functionality of PBNK. In contrast to PBNK, UCB-NK have the ability to overcome resistance to cytolysis due to HLA-ABC expression as demonstrated by the correlative studies with all ten cell lines which revealed efficient UCB-NK-mediated cytolysis of both HLA-ABC high- and low-expressing cell lines (Fig. 4c). A lack of expression of inhibitory KIRs on UCB-NK may provide a mechanistic explanation for their ability for HLA class I independent cytotoxicity. Indeed, whereas PBNK and UCB-NK expressed similar levels of NKG2A, inhibitory KIRs, as measured by a panKIR2D antibody, were completely lacking from the UCB-NK cell surface. In keeping with this observation, we previously published the profiling of UCB-NK using an expanded panel of antibodies to inhibitory KIR, which revealed low expression levels of KIR2DL1/DS1, KIR2DL2/DL3/DS2 and KIR3DL1/DS1 as compared to PBNK [32]. Cervical tumors have been shown to also have aberrant non-classical HLA class I expression which might help them to escape from NK cell killing (Heeren et al. 2016, submitted). Remarkably, in our hands, NK cytotoxicity was not impaired by higher levels of HLA-E or HLA-G expression. The apparent ability of UCB-NK to overcome the possible resistance related to expression of both inhibitory classical and non-classical HLA molecules may offer an excellent treatment modality for cervical cancer.

NK cells are often dysfunctional and low in number in cervical cancer patients [18, 22, 23]. In order to achieve a more potent and effective cytotoxic effect of NK cells in patients with cervical cancer, it is therefore critical to have adequate numbers of functional effector NK cells. In regard to generating large numbers of NK cells for therapeutic purposes, NK cells expanded from PBMC and other sources have limited expansion capacity as compared to cord blood-derived NK cells [49]. Adoptive transfer of large numbers of cytotoxic UCB-NK could be a viable treatment option, because UCB-NK have a highly activated phenotype with more than 75 % stable expression rates of NKG2D, DNAM-1, NKp30, NKp44 and NKp46 in all mature UCB-NK, and lack inhibitory KIRs, resulting in HLA-independent cytolytic efficacy; additional advantages of UCB-NK over PBNK are fewer impurities (such as T and B cells) detected upon full NK maturation, thereby reducing chances of GVHD upon adoptive transfer [30, 36]. In this study, UCB-NK were not tested in combination with CET due to their low surface expression of CD16a in vitro; however, UCB-NK further mature upon adoptive transfer in vivo which is accompanied by an increase in CD16a expression [50], and this feature could be exploited to enhance tumor killing even more via ADCC using CET and other IgG_1_ therapeutic antibodies. To facilitate clinical application, a GMP-based NK cell expansion and differentiation protocol has already been established, approved by regulatory authorities and applied in a Phase-I clinical trial for elderly acute myeloid leukemia patients and numbers of over 30 × 10^6^/kg body weight cytotoxic UCB-NK (oNKord^®^) can easily be achieved for therapeutic purposes (CCMO no NL31699 and Dutch trial register no 2818). Therefore, it is now entirely feasible to develop clinical protocols to explore, for the first time, adoptive transfer of UCB-NK in patients with solid tumors like cervical cancer.

In conclusion, our data provide a clear rationale for the use of UCB-NK to treat cervical tumors and also the possibility of using PBNK in combination with CET for EGFR-expressing tumors, with both significantly higher cytotoxicity and degranulation levels than in PBNK only conditions. Notably, treatment with UCB-NK might serve as a generally applicable treatment for cervical cancer enabled by HLA-, histology- and HPV-independent killing mechanisms.

## Electronic supplementary material

Below is the link to the electronic supplementary material.

Supplementary material 1 (PDF 722 kb)

## Abbreviations

7AAD: 7-Aminoactinomycin D
ADCC: Antibody-dependent cell-mediated cytotoxicity
CET: Cetuximab
DNAM-1: DNAX accessory molecule-1
KIR2D: Killer-cell immunoglobulin-like receptor 2D
MICA/B: Major histocompatibility complex class I polypeptide-related sequence A/B
NKG2A/C/D: Natural killer group 2, member A/C/D receptor
PBNK: Peripheral blood natural killer cells
PVR: Polio virus receptor
UCB-NK: Umbilical cord blood natural killer cells
ULBP: UL16 binding proteins
VU: Vrije Universiteit
