# Supplementary material for: High-efficiency lysis of cervical cancer by allogeneic NK cells derived from umbilical cord progenitors is independent of HLA status
Source: Cancer Immunol Immunother. 2016 Oct 25;66(1):51–61. doi: 10.1007/s00262-016-1919-1 (PMC5222919; doi:10.1007/s00262-016-1919-1)
Supplement: Supplementary file 1 — Supplementary material 1 (PDF 722 kb) [file 262_2016_1919_MOESM1_ESM.pdf]

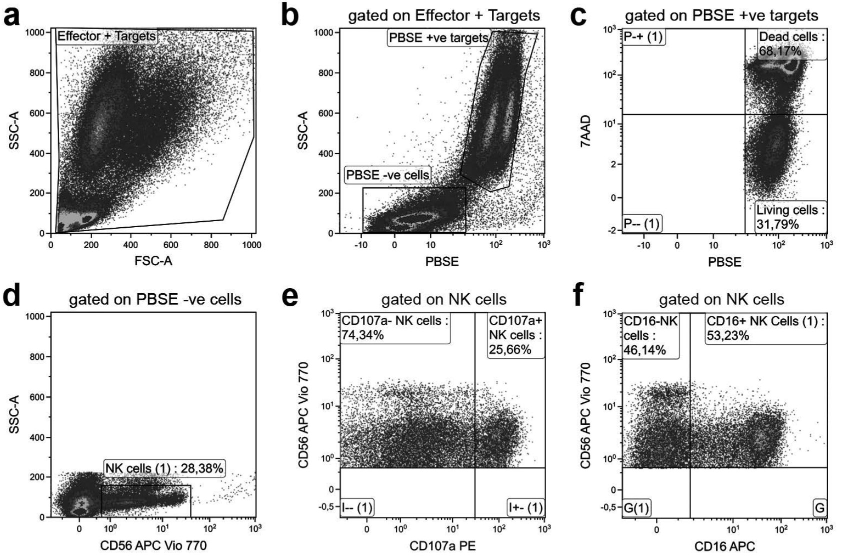

Supplementary Figure 1: Representative example of flowcytometric analysis

A representative example of flowcytometric analysis of Natural Killer (NK) cytotoxicity assay with cell line CC10A. **(a)** Identification of effectors and targets using SSC and FSC gating. **(b)** Target cells stained with PBSE were gated out from effector cells and cell debris. **(c)** PBSE-positive target cells were then gated against 7AAD to determine the percentage of cell death induced by NK cells. **(d)** Cells staining negative for PBSE and low on SSC were gated against CD56 marker to identify NK cells, further on NK cells, the levels of **(e)** NK degranulation (CD107a) and **(f)** NK $CD16^{+}$ cells percentages were calculated and compared with respective target only controls.

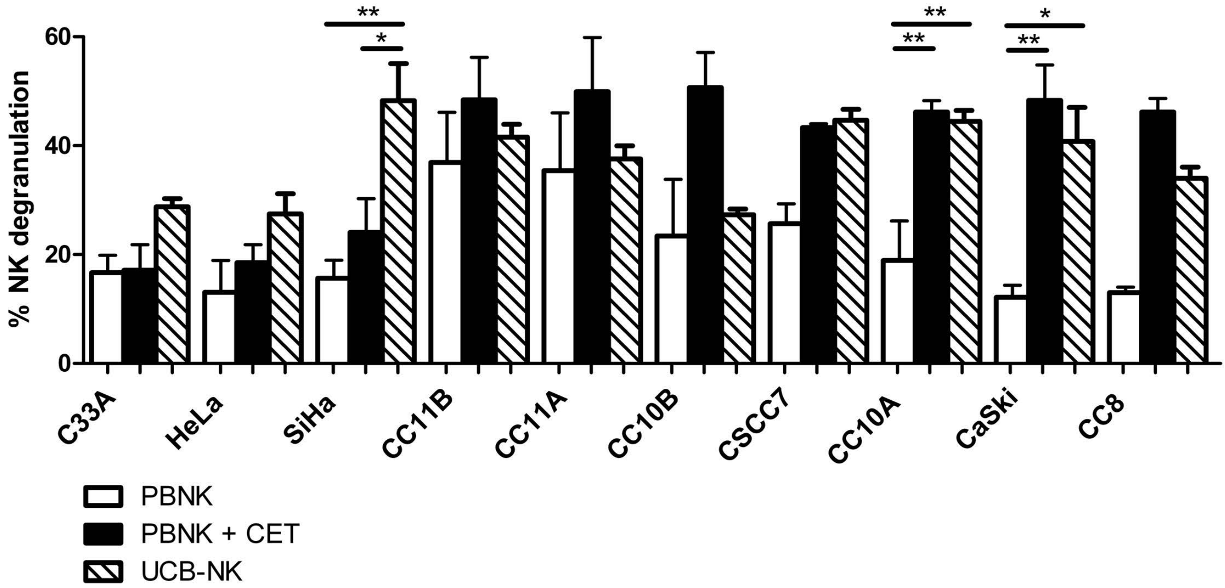

Supplementary Figure 2: NK degranulation levels per cell line for PBNK, PBNK + CET, and UCB-NK.

Significantly higher levels of NK degranulation (ΔCD107a) in PBNK + cetuximab (CET) and UCB-NK conditions compared to PBNK only condition for SiHa, CC10A and CaSki. Bars are means of triplicate values from four experiments with four different donors for C33A, HeLa, SiHa, CC11B, CC11A, CC10B, CC10A, CaSki and two experiments with two different donors for CSCC7 and CC8 using PBNK and five experiments for UCB-NK using five different donors for all cell lines. * $P < 0.05$ and ** $P < 0.01$ calculated with one-way ANOVA, Bonferroni's multiple comparison test.

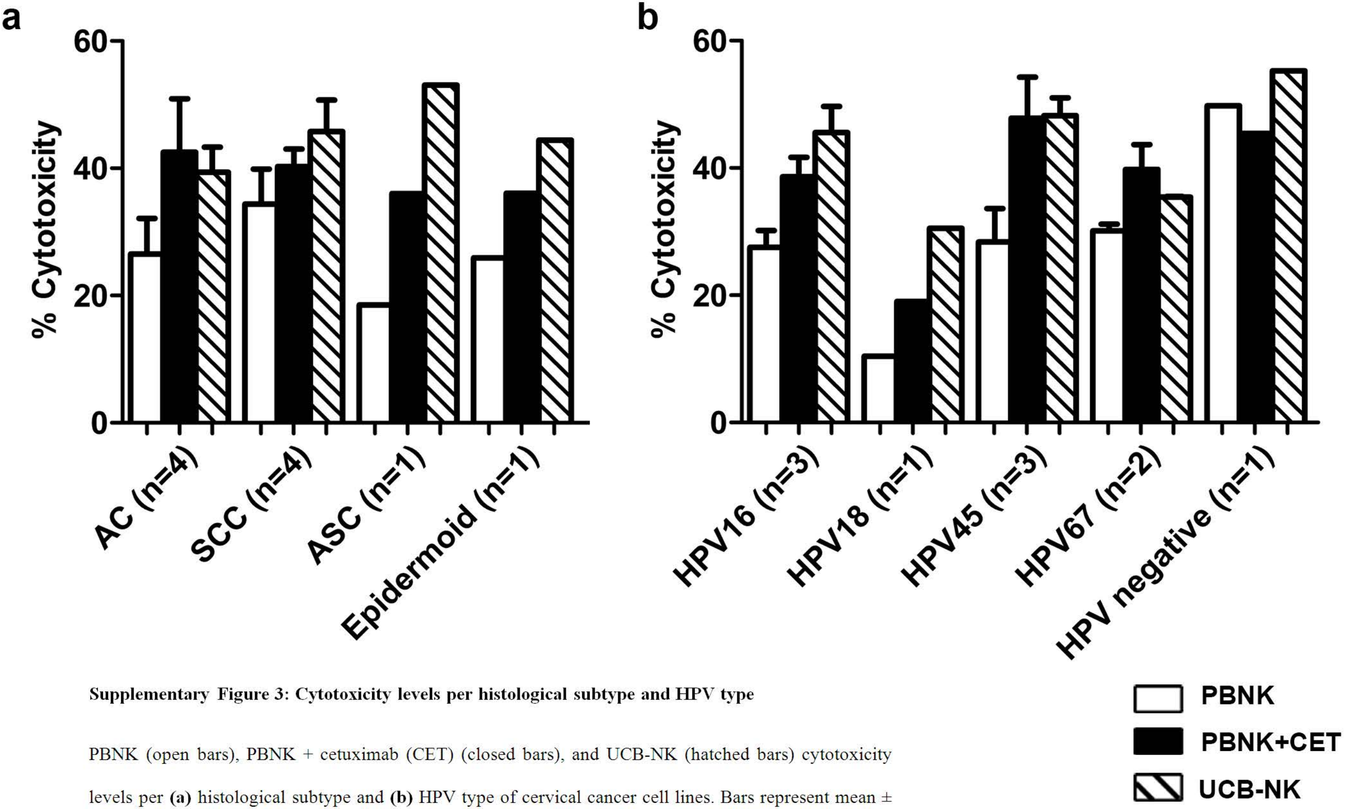

Supplementary Figure 3: Cytotoxicity levels per histological subtype and HPV type

PBNK (open bars), PBNK + cetuximab (CET) (closed bars), and UCB-NK (hatched bars) cytotoxicity levels per **(a)** histological subtype and **(b)** HPV type of cervical cancer cell lines. Bars represent mean ± SEM. Four experiments with four different PBNK donors for C33A, HeLa, SiHa, CC11B, CC11A, CC10B, CC10A, CaSki, two experiments with two different PBNK donors for CSCC7 and CC8, and five experiments with five different UCB-NK donors were used for this experiment. AC: adenocarcinoma; SCC: squamous cell carcinoma; ASC: adenosquamous cell carcinoma.
